# Clinical characteristics and predictors of complications and mortality in hospitalized octogenarian patients with COVID-19: an ambispective study

**DOI:** 10.1007/s41999-024-01063-1

**Published:** 2024-10-19

**Authors:** Marta Arroyo-Huidobro, Natàlia Pallarès Fontanet, Cristian Tebé Cordomí, Antonella F. Simonetti, Carlos Pérez-López, Gabriela Abelenda-Alonso, Alexander Rombauts, Isabel Oriol Bermudez, Elisenda Izquierdo, Vicente Díaz-Brito, Gemma Molist, Guadalupe Gómez Melis, Sebastian Videla, Alfons López Soto, Jordi Carratalà, Alejandro Rodriguez-Molinero, On behalf of MetroSud and Divine Study Group, Carlota Gudiol, Judit Aranda-Lobo, Montserrat Sanmartí, Encarna Moreno, Maria C. Alvarez, Ana Faura, Martha González, Paula Cruz, Mireia Colom, Andrea Perez, Laura Serrano, Sebastià Videla, Mireia Besalú, Erik Cobo, Jordi Cortés, Daniel Fernández, Leire Garmendia, Guadalupe Gómez, Pilar Hereu, Klaus Langohr, Núria Pérez-Álvarez, Xavier Piulachs, Natàlia Pallares, Cristian Tebé, Mireia Besalú, Erik Cobo, Jordi Cortés, Daniel Fernández, Klaus Langohr, Núria Pérez-Álvarez, Xavier Piulachs, Guadalupe Gómez

**Affiliations:** 1 Geriatric Unit, Hospital Clinic de Barcelona, C. de Villarroel, 170, 08036 Barcelona, Spain; 2 Biostatistics Support and Research Unit, Germans Trias i Pujol Research Institute and Hospital (IGTP), Badalona, Catalunya Spain; 3 Infectious Diseases Unit, Hospital de la Santa Creu i Sant Pau, Barcelona, Catalunya Spain; 4 Consorci Sanitari Alt Pènedes I Garraf, Area de Recerca, Barcelona, Catalunya Spain; 5 Department of Infectious Diseases, Bellvitge University Hospital, L’Hospitalet de Llobregat, Catalunya Spain; 6 Department of Internal Medicine, Consorci Sanitari Integral, L’Hospitalet de Llobregat, Catalunya Spain; 7 Department of Anaesthesiology, Hospital de Viladecans, Viladecans, Catalunya Spain; 8 Parc Sanitari Sant Joan de Déu, Sant Boi de Llobregat, Spain; 9 Biostatistics Unit of the Bellvitge Biomedical Research Institute (IDIBELL), L’Hospitalet de Llobregat, Catalunya Spain; 10 Department of Statistics and Operations Research, Universitat Politècnica de Catalunya/Barcelonatech, Barcelona, Catalunya Spain; 11 Department of Clinical Pharmacology, Bellvitge University Hospital, L’Hospitalet de Llobregat, Catalunya Spain; 12 Hospital Clinic de Barcelona, Geriatric Unit, Department of Internal Medicine, Institut d’Investigacions Biomèdiques August Pi i Sunyer (IDIBAPS), Barcelona, Catalunya Spain; 13 Consorci Sanitari Alt Penedes-Garraf, Area de Recerca, Barcelona, Catalunya Spain

**Keywords:** COVID-19, Octogenarians, Clinical complications, Mortality, Risk factors, Pandemic waves, Aged 80 and over

## Abstract

**Objective:**

This study aimed to describe the clinical presentation of COVID-19 in hospitalized patients aged 80 or above and to identify predictors for death and complications throughout the epidemic waves of the disease.

**Methods:**

This was an observational, multicenter, ambispective study conducted between March 2020 and August 2021 using data collected in five centers from southern metropolitan area of Barcelona (COVID-MetroSud cohort). Patients were grouped based on the pandemic waves of inclusion in the registry. We conducted a descriptive analysis, followed by bivariate and multivariate analyses (binary logistic regression) to identify predictors of risk for death or complications.

**Results:**

A total of 1192 patients (mean [SD] age 85.7 [4.22] years and 46.8% female) were included. The most frequently reported symptoms in all waves were fever (63.1%), cough (56.5%), dyspnea (48.2%), and asthenia (27.5%). Laboratory and radiological findings consistently showed abnormal bilateral chest X-ray results (72.5% of patients) and elevated inflammatory markers such as lactate dehydrogenase (mean [SD] 335 [188] U/L), C-reactive protein (CRP) (mean [SD] 110 [88.4] U/L), and ferritin (mean [SD] 842 [1561] U/L). Acute respiratory distress syndrome (43.7%), renal failure (19.2%), and delirium (17.5%) were the most frequent complications. The overall mortality rate was 41.4% and declined across the epidemic waves. Age, diabetes mellitus, heart failure, dyspnea, and higher baseline levels of creatinine were identified as risk factors for complications, while a higher Barthel index and presence of cough were found to be protective. Age, dyspnea, abnormal bilateral chest X-ray, CRP, and sodium were identified as risk factors for death.

**Conclusions:**

This study demonstrates the clinical presentation of COVID-19 (fever, cough, dyspnea, and asthenia) and the different risk factors for mortality and complications in octogenarian hospitalized patients throughout the pandemic. These findings could be highly valuable for managing future virus pandemics.

**Supplementary Information:**

The online version contains supplementary material available at 10.1007/s41999-024-01063-1.

## Key summary points

**Aim:**

This the study describes the clinical presentation of COVID-19 and the risk factors for complications and death in octogenarian hospitalized patients across the different waves of the disease.

**Findings:**

The most frequently reported symptoms in hospitalized octogenarian patients were fever, cough, dyspnea, and asthenia with acute respiratory distress syndrome, renal failure, and delirium being the most frequent complications. Regarding complications, diabetes mellitus, heart failure, dyspnea, and higher baseline levels of creatinine were identified as risk factors, while a higher Barthel index and presence of cough were found to be protective. Age, dyspnea, abnormal bilateral chest x-ray, CRP, and sodium were identified as risk factors for death.

**Message:**

These findings could be valuable for managing future pandemics by contributing to tailored interventions and strategies to reduce COVID-19 mortality and complications in this patient group.

**Supplementary Information:**

The online version contains supplementary material available at 10.1007/s41999-024-01063-1.

## Introduction

The SARS-CoV-2 pandemic (COVID-19) had a significant impact on healthcare systems worldwide. Since its spread in March 2020 to the present day 765 million cases and 6.9 million deaths have been recorded worldwide [1]. In Spain, since the start of the pandemic, 13.8 million cases and 120,964 deaths have been reported, of which 931,616 cases and 77,136 deaths were among patients over 80 years of age [2, 3].

Older individuals experience physiological changes associated with aging, multiple age-related comorbidities, and polypharmacy [4]. The immune system of older adults undergoes various age-related changes, collectively known as immune senescence, which affect both the innate and adaptive immune systems. As a result, this population becomes more susceptible to infectious diseases [5]. These factors contribute to the occurrence of nonspecific and atypical clinical manifestations of COVID-19 [6].

Additionally, age is one of the most important factors associated with mortality in patients with COVID-19 admitted to the hospital [7, 8]. Early statistical data from China reported a mortality rate in people over 80 years of up to 14.8% [9, 10]. Similarly, a meta-analysis reported the highest mortality rates in this age segment, which were six times higher than in younger patients [11]. The increased mortality rates and complications in older patients may be attributed to a high multimorbidity, including dementia [12] and geriatric syndromes, increased frailty, and lower physiological and functional reserve [13].

Various epidemiological studies have described COVID-19 and identified risk factors for mortality in the general population. These factors include underlying pathologies, such as hypertension, cardiovascular disease, diabetes, chronic obstructive pulmonary disease, and cancer, presenting symptoms, such as dyspnea and expectoration, and radiological or analytical data, such as lactate dehydrogenase, D-dimer, and troponin [14–17]. However, limited epidemiological studies have investigated the clinical characteristics of COVID-19 in the very older population, only a few have identified risk factors for mortality, and none have focused on risk factors for complications in this population across multiple epidemics waves of the disease.

Considering Spain’s significant proportion of older individuals, with 6.2% of the population aged 80 or older, and the limited information available regarding patient profiles and clinical outcomes for this age group across different waves of the pandemic, there is a crucial need to gain a comprehensive understanding of COVID-19 in this specific population. This ambispective study aimed to describe the clinical presentation of COVID-19 in hospitalized patients aged 80 or above using data collected in five centers from the region south of Barcelona, Spain, throughout the epidemic waves of the disease. In addition, we aimed to identify predictors for death and complications in this population.

## Methods

### Study design and population

This was an observational, multicenter, ambispective study conducted between March 2020 and August 2021 in the southern metropolitan area of Barcelona, Spain. The data were collected from electronic medical records obtained from the COVID-MetroSud cohort, which comprises five large healthcare centers in the region: Hospital Universitario de Bellvitge (a tertiary-level hospital), Complejo Hospitalario Moisés Broggi, Consorci Sanitari Alt Penedès i Garraf, Hospital Viladecans, and Parc Sanitari Sant Joan de Déu (secondary-level hospitals). These healthcare centers collectively serve a reference population of 1,200,000 inhabitants and have a total of 2000 beds, including over 150 intensive care beds (expanded to 200 during the peak of the COVID-19 epidemic) and 200 intermediate care beds.

Patients were grouped based on the pandemic waves of inclusion in the registry. The first wave spanned from March 1 to March 15, 2020, the second wave from October 1 to November 30, 2020, the third wave from January 1 to February 28, 2021, the fourth wave from April 2021 1 to May 31, 2021 and the fifth wave from July 1 to August 31, 2021. No data were recorded for the fourth epidemic wave, which was of lower intensity and shorter duration than the others.

This study included hospitalized patients aged ≥ 80 years who were diagnosed with COVID-19 using real-time reverse transcription polymerase chain reaction (rRT-PCR) tests on nasal or pharyngeal samples. Patients with respiratory symptoms compatible with COVID-19 who were treated upon admission but had negative rRT-PCR tests were excluded. Additionally, patients who were not admitted, either because they were transferred to other hospitals or died in the emergency department, were also excluded.

Patient data were collected in real-time from the hospital information service in the REDCap (Research Electronic Data Capture) platform version 10.7.0, from admission with a prospective follow-up until death, discharge, transfer to another center (August 2021) [18, 19]. Data from deceased and discharged patients at the time of inclusion were retrospectively recorded.

Structured information (i.e., demographic data and laboratory results) was extracted from hospital electronic medical records for analysis. Unstructured information contained in the clinical course of patients was extracted by investigators from different participating centers using an electronic case report form implemented in the REDCap platform version 10.7.0 [18, 19].

For pseudonymization, all study data were identified by a case code and stored without any accompanying personal identification data. Considering that routinely collected data were anonymized, informed consent was not required. The sponsor and investigators of the study ensured that patient data procedures followed the General Data Protection Regulation (EU) 2016/679 (GDPR) and the Organic Law 3/2018 of December 5 on the Protection of Personal Data and guarantee of digital rights in Spain. This study was approved by the Research Ethics Committee of the Hospital Universitario de Bellvitge. Additionally, it was developed in accordance with ethical principles originating from the latest version of the Helsinki Declaration accepted by local authorities and in line with Good Clinical Practice (GCP) and the requirements of current Spanish regulations.

### Endpoints and variables

The main objective of this study was to describe the clinical presentation of COVID-19 in hospitalized octogenarian patients throughout the epidemic waves of the disease. As secondary objectives, we evaluated the complications presented by older patients hospitalized due to COVID-19 and identified predictors for complications and mortality.

At the time of admission, variables related to the form of presentation of the disease were recorded, including presentation symptoms (e.g., presence or absence of cough, fever, dyspnea), vital signs (heart rate, blood pressure, and oxygen saturation), pulmonary auscultation data (presence of pathological sounds and type). At the time of disease presentation, the degree of radiological involvement (none, unilateral, or bilateral), all available laboratory data, and the appearance of clinical complications (ARDS, delirium, cardiac and renal complications) were also recorded. (Table S3).

In addition, we collected for each patient sociodemographic (date of birth, sex, age, institutionalization), and clinical data including Barthel index score (a scoring system that measures a patient’s performance in activities of daily living ranging from 0: dependent to 100: independent), toxic habits (smoker/alcoholic), body mass index (BMI), previous pathologies, degree of comorbidity according to the Charlson Index, and previous pharmacological treatment, including antiplatelet and anticoagulant drugs (active ingredient and dosage), anti-inflammatory drugs, antidiabetic drugs, and cardiovascular, respiratory, and immune system medications).

### Statistical analysis

A descriptive analysis of all study variables was performed. Categorical variables were expressed as frequencies and percentages (%) and continuous variables were described using mean and standard deviation (SD), or median and interquartile range [IQR].

To identify predictors of risk for death or developing complications, we constructed several multivariate models (binary logistic regression), including variables significantly associated with the outcome in a previous bivariate analysis (*p* < 0.05) and those with clinical relevance according to the researchers' criteria. The independent variables were analyzed in blocks: (1) sociodemographic variables and pathological antecedents, (2) variables related to clinical presentation, and (3) laboratory findings. All models were adjusted for age and sex. Similarly, multivariate models were constructed to identify predictors of additional outcomes: cardiac complications, renal complications, and delirium. All estimates were reported with their odds ratio (OR) and 95% confidence interval (CI).

The database was reviewed to remove erroneous data and outliers as evaluated by the investigator. Patients with missing data for any of the following variables were excluded: age, sex, comorbidities evaluated in Charlson Index Score or outcome (discharge, exitus). Statistical analysis was performed using R version 4. 1.0 (R Core Team 2021).

## Results

### Study population and characteristics

**Fig. 1:**
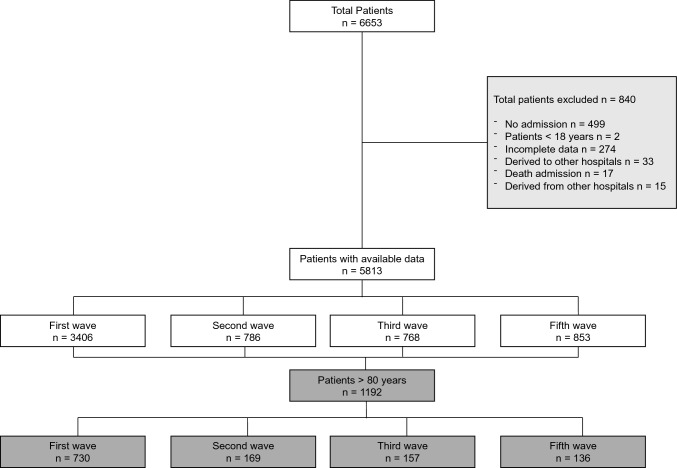
Patient selection flowchart

A total of 6653 patients diagnosed with COVID-19 were included in the MetroSud registry, of which 1192 were 80 or older and were included in this study. Of these, 61.2% (*n* = 730) were admitted in the first epidemic wave of COVID-19 and 38.8% (*n* = 462) in successive waves (Fig. 1).

Patients included had a mean age of 85.7 years (SD 4.22) and 46.8% were female. Most patients were non-smokers, did not reside in nursing homes, were overweight or obese, and had hypertension and dyslipidemia. The mean Barthel index was 78.2 (SD 28.9) and the Charlson comorbidity index was 6.23 (SD 1.84) (Table 1). Regarding previous treatments (described in Table S1), the most frequently prescribed medications were statins, ACE inhibitors, acetylsalicylic acid, and antidiabetic drugs. There were two vaccinated patients in the third wave (1.3%) and 121 (89.0%) in the fifth wave (Tables 1, 2).

**Table 1:** Sociodemographic and clinical characteristics

	1st wave *n* = 730	2nd wave *n* = 169	3rd wave *n* = 157	5th wave *n* = 136	Total *n* = 1192
Sociodemographic characteristics					
Sex, *n* (%)					
Male	394 (54.0)	88 (52.1)	80 (51.0)	72 (52.9)	634 (53.2)
Female	336 (46.0)	81 (47.9)	77 (49.0)	64 (47.1)	558 (46.8)
Age (years), *mean *(*SD*)	85.4 (4.04)	86.0 (4.47)	85.9 (4.36)	86.9 (4.51)	85.7 (4.22)
Smoker, *n* (%)					
No	555 (76.0)	125 (74.0)	120 (76.4)	90 (66.2)	890 (74.7)
Yes	13 (1.78)	5 (2.96)	5 (3.18)	5 (3.68)	28 (2.35)
Ex-smoker	162 (22.2)	39 (23.1)	32 (20.4)	41 (30.1)	274 (23.0)
Alcohol consumption, *n* (%)	10 (1.37)	9 (5.33)	9 (5.73)	2 (1.47)	30 (2.52)
Long-term facility, *n* (%)	193 (26.4)	19 (11.2)	18 (11.5)	10 (7.35)	240 (20.1)
Complete COVID-19 vaccine	0 (0%)	0 (0%)	2 (1.29%)	121 (89,0%)	123 (10.3%)
Categorical BMI (kg/m^2^), *n* (%)					
Low weight	2 (0.41)	1 (0.68)	0 (0.00)	0 (0.00)	3 (0.33)
Normal	96 (19.7)	29 (19.6)	25 (17.9)	31 (25.4)	181 (20.2)
Overweight	248 (50.8)	68 (45.9)	57 (40.7)	47 (38.5)	420 (46.8)
Obese	142 (29.1)	50 (33.8)	58 (41.4)	44 (36.1)	294 (32.7)

**Table 2:** Comorbidities

	1st wave *n* = 730	2nd wave *n* = 169	3rd wave *n* = 157	5th wave *n* = 136	Total *n* = 1192
Diabetes mellitus, *n* (%)	245 (33.6)	55 (32.5)	52 (33.1)	45 (33.1)	
With target organ involvement	73 (29.8)	12 (21.8)	0 (0.00)	0 (0.00)	85 (21.4)
Without complications	172 (70.2)	43 (78.2)	52 (100)	45 (100)	312 (78.6)
Chronic pulmonary pathology, *n* (%)	192 (26.3)	51 (30.2)	36 (22.9)	43 (31.6)	322 (27.0)
COPD	110 (57.3)	22 (43.1)	15 (41.7)	28 (65.1)	175 (54.3)
OSA	50 (16.9)	18 (35.3)	7 (19.4)	6 (14.0)	81 (19.0)
Asthma	39 (13.2)	13 (25.5)	8 (22.2)	7 (16.3)	67 (15.7)
Interstitial pneumopathy	7 (3.65)	3 (5.88)	6 (16.7)	5 (11.6)	21 (6.52)
Hypertension, *n* (%)	571 (78.2)	136 (80.5)	125 (79.6)	121 (89.0)	953 (79.9)
Dyslipidemia, *n* (%)	327 (44.8)	93 (55.0)	93 (59.2)	72 (52.9)	585 (49.1)
Moderate-severe kidney disease, *n* (%)	103 (14.1)	19 (11.2)	10 (6.37)	4 (2.94)	136 (11.4)
Hemodialysis, *n* (%)	9 (1.23)	2 (1.18)	1 (0.64)	2 (1.47)	14 (1.17)
Heart failure, *n* (%)	150 (20.5)	28 (16.6)	29 (18.5)	32 (23.5)	239 (20.1)
Coronary heart disease, *n* (%)	81 (11.1)	29 (17.2)	25 (15.9)	16 (11.8)	151 (12.7)
Acute myocardial infarction, *n* (%)	81 (11.1)	16 (9.47)	14 (8.92)	14 (10.3)	125 (10.5)
Atrial fibrillation, *n* (%)	137 (18.8)	44 (26.0)	38 (24.2)	43 (31.6)	262 (22.0)
Hematologic malignancy, *n* (%)	10 (1.37)	8 (4.73)	4 (2.55)	14 (10.3)	36 (3.02)
Non-metastatic malignancy, *n* (%)	71 (9.73)	22 (13.0)	19 (12.1)	26 (19.1)	138 (11.6)
Ictus, *n* (%)	112 (15.3)	23 (13.6)	24 (15.3)	22 (16.2)	181 (15.2)
Ischemic or hemorrhagic stroke with sequelae	29 (38.7)	9 (39.1)	7 (29.2)	5 (22.7)	50 (34.7)
TIA or ischemic stroke without sequelae	46 (61.3)	14 (60.9)	17 (70.8)	17 (77.3)	94 (65.3)
Peripheral vasculopathy, *n* (%)	50 (6.85)	23 (13.6)	19 (12.1)	11 (8.09)	103 (8.64)
Dementia, *n* (%)	173 (23.7)	44 (26.0)	31 (19.7)	33 (24.3)	281 (23.6)
Anxiety, *n* (%)	31 (4.25)	14 (8.28)	13 (8.28)	6 (4.41)	64 (5.37)
Depression, *n* (%)	67 (9.18)	25 (14.8)	23 (14.6)	12 (8.82)	127 (10.7)
Connective tissue disease, *n* (%)	37 (5.07)	13 (7.69)	14 (8.92)	11 (8.09)	75 (6.29)
Rheumatoid arthritis	7 (22.6)	5 (38.5)	0 (0.00)	6 (54.5)	18 (26.1)
SLE	1 (3.23)	0 (0.00)	0 (0.00)	1 (9.09)	2 (2.90)
Other	23 (74.2)	8 (61.5)	14 (100)	4 (36.4)	49 (71.0)
Neurodegenerative disease, *n* (%)	35 (4.79)	14 (8.28)	8 (5.10)	3 (2.21)	60 (5.03)
Barthel Index, *mean *(*SD*)	75.7 (31.8)	79.0 (26.6)	83.4 (23.8)	79.6 (25.7)	78.2 (28.9)
Charlson Index, *mean *(*SD*)	6.22 (1.85)	6.43 (2.10)	5.99 (1.63)	6.27 (1.58)	6.23 (1.84)

*SD* standard deviation, *BMI* body mass index, *DM* diabetes mellitus, *COPD* chronic obstructive pulmonary disease, *OSA* obstructive sleep disease, *TIA* transient ischemic attack, *SLE* systemic lupus erythematosus

### Clinical presentation of COVID-19

The most common recorded symptoms were fever (63.1%), cough (56.5%), dyspnea (48.2%), and asthenia (27.5%) (Table 3). The overall incidence of delirium was 11.0% and was higher during the initial two waves of the epidemic than the subsequent two waves. Rhinorrhea and headache incidence were higher in third and fifth waves. In the physical examination findings (Table 4), as the epidemic waves progressed, patients presented hypoxemia less frequently and better respiratory rate and SAFI (oxygen saturation [SpO2]/fraction of inspired oxygen [FiO2] ratio).

**Table 3:** Most frequent patient’s symptoms at admission (> 5% of patients), *n* (%)

	1st wave *n* = 730	2nd wave *n* = 169	3rd wave *n* = 157	5th wave *n* = 136	Total *n* = 1192
Rhinorrhea	23 (3.15)	5 (2.96)	7 (4.46)	16 (11.8)	51 (4.28)
Anosmia	14 (1.92)	11 (6.51)	11 (7.01)	5 (3.68)	41 (3.44)
Ageusia	25 (3.42)	11 (6.51)	14 (8.92)	6 (4.41)	56 (4.70)
Arthromyalgia	133 (18.2)	32 (18.9)	12 (7.64)	15 (11.0)	192 (16.1)
Fever	495 (67.8)	101 (59.8)	80 (51.0)	76 (55.9)	752 (63.1)
Chills	48 (13.3)	9 (8.91)	4 (5.00)	3 (3.95)	64 (10.3)
Cough	415 (56.8)	91 (53.8)	86 (54.8)	82 (60.3)	674 (56.5)
Dyspnea	359 (49.2)	81 (47.9)	73 (46.5)	62 (45.6)	575 (48.2)
Expectoration	116 (15.9)	26 (15.4)	21 (13.4)	27 (19.9)	190 (15.9)
Diarrhea	135 (18.5)	34 (20.1)	38 (24.2)	29 (21.3)	236 (19.8)
Vomiting	42 (5.75)	8 (4.73)	7 (4.46)	8 (5.88)	65 (5.45)
Nausea	49 (6.71)	9 (5.33)	5 (3.18)	7 (5.15)	70 (5.87)
Asthenia	168 (23.0)	59 (34.9)	62 (39.5)	39 (28.7)	328 (27.5)
Anorexia	79 (10.8)	26 (15.4)	33 (21.0)	21 (15.4)	159 (13.3)
Headache	25 (3.42)	9 (5.33)	17 (10.8)	15 (11.0)	66 (5.54)
Chest pain	24 (3.29)	9 (5.33)	6 (3.82)	3 (2.21)	42 (3.52)
Abdominal pain	24 (3.29)	10 (5.92)	9 (5.73)	6 (4.41)	49 (4.11)
Delirium*	85 (11.6)	28 (16.6)	9 (5.73)	9 (6.62)	131 (11.0)
Bacterial infection	17 (2.33)	3 (1.78)	0 (0.00)	1 (0.74)	21 (1.76)
Previous visit to the emergency room	71 (9.73)	21 (12.4)	14 (8.92)	12 (8.82)	118 (9.90)

*Delirium was defined as any acute alteration in cognition or inattention in the emergency report or at the time of admission

**Table 4:** Physical examination findings

	1st wave *n* = 730	2nd wave *n* = 169	3rd wave *n* = 157	5th wave *n* = 136	Total *n* = 1192
Temperature ºC, *mean *(*SD*)	36.9 (1.02)	36.7 (0.88)	36.6 (0.81)	36.7 (0.84)	36.8 (0.96)
Provided FiO2%, *mean *(*SD*)	28.4 (18.6)	28.7 (16.5)	30.2 (18.5)	28.9 (14.3)	28.7 (17.8)
SBP mmHg, *mean *(*SD*)	131 (23.6)	136 (26.1)	137 (24.5)	135 (23.6)	133 (24.2)
DBP mmHg, *mean *(*SD*)	70.2 (14.9)	70.9 (12.8)	74.5 (13.1)	69.5 (13.2)	70.8 (14.3)
Oxygen saturation %, *mean *(*SD*)	92.1 (6.89)	93.6 (4.83)	94.5 (4.91)	95.0 (3.40)	93.0 (6.16)
Heart rate BPM, *mean *(*SD*)	87.3 (18.2)	83.3 (18.1)	84.1 (18.6)	81.5 (16.4)	85.6 (18.1)
Respiratory rate, BPM, *mean *(*SD*)	22.9 (6.91)	22.8 (6.24)	22.3 (5.99)	20.4 (4.82)	22.5 (6.47)
SAFI, *mean *(*SD*)	386 (104)	380 (103)	371 (107)	369 (94.4)	381 (103)
Normal respiratory exploration, *n* (%)	100 (18.6)	26 (16.6)	23 (14.7)	15 (11.0)	164 (16.6)
Abnormal respiratory exploration, *n* (%)	373 (86.3)	114 (87.0)	118 (88.7)	107 (88.4)	712 (87.1)
Sibilants	50 (12.3)	20 (15.3)	11 (8.27)	20 (16.5)	101 (12.8)
Roncus	74 (17.8)	27 (20.6)	14 (10.5)	16 (13.2)	131 (16.4)

*SD* standard deviation, *SBP* systolic blood pressure, *DBP* diastolic blood pressure, *BPM* beats/breaths per minute, *SAFI* oxygen saturation (SpO2)/fraction of inspired oxygen (FiO2) ratio

Radiological and laboratory findings on admission are described in Table S2. Older patients admitted for COVID-19 frequently presented leukocytosis with neutrophilia and lymphopenia. In addition, they had elevated lactate dehydrogenase (LDH) and acute phase reactants (C-reactive protein [CRP] and serum ferritin levels), and altered coagulation parameters (fibrinogen, and D-dimer) and liver and renal functions.

Mean levels of inflammatory parameters (D-dimer, CRP, LDH, and ferritin levels) decreased after the first wave. In addition, bilateral infiltrates were detected at lower frequencies after the first wave.

### Complications

Mortality and complications are described in Table S3. Of the patients included, 41.4% (*n* = 493) died. Mortality decreased progressively with each new wave, from 46.2% in the first wave to 24.3% in the fifth wave. Overall, 47.9% (*n* = 569) of patients experienced some complications. A higher prevalence of complications was observed in the first and second epidemic waves and decreased in the third and fifth waves. The most common complications were acute respiratory distress syndrome (ARDS) (43.7%), followed by renal failure (19.2%), and delirium (17.5%). Table S4 shows the results of a stratified analysis of mortality and complications separately from the first wave to the combined subsequent waves.

### Variables associated with the risk of complications and death

The multivariate analysis of risk factors associated with death and complications, including sociodemographic variables and comorbidities, showed that age was significantly associated with the risk of death, and age, male sex, and diabetes mellitus were associated with the risk of complications (Table 5). Diabetes mellitus (DM) and heart failure were associated with a 58% and 61% increase in the risk of complications, whereas, with each 5-year increment in age, the risk of complications and death increased by 22% (*p* = 0.039) and 25%, respectively (*p* = 0.024). Furthermore, 10-units increment in the Barthel index score was associated with a 7% lower risk of both complications and death (*p* = 0.012 and *p* = 0.028, respectively).

**Table 5:** Multivariate analysis assessing the association between sociodemographic, clinical and laboratory predictors with the risk of complications and death

Predictors	Risk of any complication				Risk of death			
	Odds ratio	Std. Error	95% CI	p- value	Odds Ratio	Std. Error	95% CI	*p*-value
Sociodemographic								
Age	1.22	0.12	1.01–1.48	**0.039**	1.25	0.12	1.03–1.51	**0.024**
Sex (Female)	0.65	0.11	0.47–0.90	**0.010**	0.79	0.13	0.56–1.08	0.130
Barthel index	0.93	0.03	0.87–0.99	**0.035**	0.93	0.03	0.87–0.98	**0.028**
Diabetes mellitus	1.58	0.26	1.14–2.20	**0.006**				
Hypertension	0.92	0.19	0.61–1.39	0.682	0.67	0.14	0.45–1.02	0.058
Dyslipidemia	1.13	0.18	0.82–1.55	0.455	1.32	0.21	0.96–1.81	0.093
COPD	0.69	0.13	0.48–1.00	0.053	0.98	0.19	0.67–1.43	0.931
Dementia	1.01	0.22	0.66–1.55	0.961	1.13	0.25	0.74–1.73	0.565
Corticosteroids	1.22	0.25	0.81–1.83	0.345	1.20	0.25	0.79–1.80	0.3841
Heart failure	1.61	0.30	1.12–2.33	**0.010**	1.11	0.21	0.76–1.60	0.598
Clinical								
Cough	0.83	0.12	0.62–1.11	0.217	0.58	0.09	0.43–0.78	** < 0.001**
Dyspnea	1.39	0.20	1.04–1.85	**0.025**	1.84	0.28	1.37–2.49	** < 0.001**
Abnormal unilateral chest x-ray	0.84	0.25	0.47–1.49	0.548	1.17	0.39	0.61–2.26	0.638
Abnormal bilateral chest x-ray	0.86	0.20	0.55–1.35	0.504	2.16	0.56	.31–3.65	**0.003**
Delirium					2.77	0.67	1.73–4.49	** < 0.001**
Laboratory								
Total leukocytes	1.07	0.23	0.70–1.62	0.751	1.16	0.25	0.75–1.78	0.496
D- Dimer	1.00	0.00	1.00–1.00	**0.015**	1.00	0.00	1.00–1.00	0.077
CRP	1.02	0.01	1.00–1.04	0.060	1.07	0.01	1.05–1.09	** < 0.001**
Sodium	1.15	0.15	0.89–1.50	0.285	1.80	0.25	1.37–2.40	** < 0.001**
Creatinine	2.45	0.41	1.78–3.63	** < 0.001**	1.34	0.21	0.99–1.84	0.061

Age (continuous variable) was evaluated as 5-year increment. Barthel Index was evaluated as 10 units increment. Total leukocytes, CRP, sodium, and creatinine were evaluated as 10 units increments. Ddimer was evaluated as 50 units increments. Bold values indicate significant findings

*Std* standard, *95% CI* 95% confidence interval, *COPD* chronic obstructive pulmonary disease, *CRP* C-reactive protein

In multivariate models that considered clinical variables, dyspnea was associated with an 84% increased risk of death (*p* < 0.001), an abnormal bilateral chest X-ray increased the risk of death by 116% (*p* = 0.024), and the presence of delirium increased the risk of death by 177% (*p* < 0.001). Interestingly, cough emerged as a protective factor against death, reducing the risk by 42% (*p* < 0.001). Additionally, dyspnea also increased the risk of complications by 39% (*p* = 0.024) (Table 5).

In multivariate models incorporating laboratory findings, a 10-unit increment in creatinine levels was associated with a 145% increment in the risk of complications (*p* < 0.001). Additionally, a 10-unit increase in CRP and sodium levels was associated with a 7% (*p* < 0.001) and 80% (*p* < 0.001) increase in the risk of death, respectively (Table 5).

### Variables associated with the risk of specific complications

In the multivariate analysis assessing risk factors for the development of delirium as a complication of COVID-19 (described in Table S5), female sex was identified as a protective factor, reducing the risk by 39% (*p* = 0.023). On the other hand, dementia and each 10-unit increase in sodium levels were associated with 86% (*p* < 0.019) and 45% (*p* < 0.016) increase in the risk of developing the syndrome, respectively. In terms of cardiac complications, heart failure, and dyspnea were identified as the only predictive factors, with a 170% (*p* < 0.001) and 156% (*p* < 0.001) increase in the risk of occurrence, respectively (Table S6). Finally, DM, heart failure, and each 10-unit increment in sodium levels were associated with an increase of 86% (*p* = 0.008), 67% (*p* = 0.029), and 62% (*p* < 0.002) in the risk of renal complications, respectively. Notably, each 10-unit increase in baseline creatinine levels was found to be related to an eight-fold increase in the risk of renal complications (*p* < 0.001) (Table S7).

A summary of the variables associated with death and complications is included in Table S8.

## Discussion

This study comprehensively describes the clinical presentation of COVID-19 and the risk factors for complications and death in octogenarian hospitalized patients across the different waves of the disease. Thus, the most frequently reported symptoms in all waves were fever, cough, dyspnea, and asthenia. Laboratory and radiological findings consistently showed abnormal bilateral chest X-ray results and elevated inflammatory markers such as LDH, CRP, and ferritin. Among the observed complications, ARDS, renal failure, and delirium were the most frequent. The overall mortality rate was 41.4% and declined across the epidemic waves.

In our study, age, DM, heart failure, dyspnea, and higher baseline levels of creatinine were identified as risk factors for complications, while a higher Barthel index and presence of cough were found to be protective. Similarly, age, dyspnea, delirium, abnormal bilateral chest x-ray, CRP, and sodium were risk factors for death. In terms of specific complications, dementia and sodium levels were identified as risk factors for delirium, while female sex was a protective factor. Additionally, dementia and elevated sodium levels were identified as risk factors for cardiac complications, and DM, heart failure, and elevated creatinine levels for renal complications.

The comorbidities most frequently found in our study (hypertension and dyslipidemia) are also considered common older patients without COVID-19, suggesting that these conditions may not be related to the disease [20]. However, hospitalized patients in this cohort had a higher prevalence of overweight or obesity in contrast to general older Spanish population without COVID-19 [21].

Regarding the clinical symptoms, the most frequently observed in our study (fever, cough, dyspnea, and asthenia) have also been reported as the most common symptoms in cohorts of older Spanish patients hospitalized for COVID-19 [22–24]. During the later epidemic waves, the clinical presentation changed significantly, with a higher proportion of patients presenting with rhinorrhea and headache. Respiratory parameters also improved on average in the later waves, suggesting less severe respiratory involvement. Consistent with the findings of other studies, the older individuals in our cohort predominantly presented elevated inflammatory markers, such as ferritin and CRP, and frequent elevation of D-dimer and LDH [22, 23]. This laboratory profile did not differ across the various epidemic waves.

One in ten patients in our sample presented with delirium at admission, with a higher incidence during the initial two waves of the epidemic compared to the subsequent two, similar to that described by Minnema [25]. The decrease in clinical severity among hospitalized older patients in each new wave could explain the decreasing incidence. Furthermore, the relaxation of hospital regulations in later waves allowed companions in patient rooms. Nevertheless, delirium is frequently underdiagnosed and underreported, suggesting its actual incidence during COVID-19 could be higher than detected in our study [26]. Moreover, the methods used for delirium detection vary across studies, lacking rigor, leading to highly heterogeneous reported frequencies. Gutiérrez-Rodríguez and Annweiler reported a similar frequency of delirium to our study in the subgroup of patients aged 80 or older, while other authors found higher frequencies [22, 23, 27–29].

A decrease in the prevalence of complications was observed in the third and fifth waves possibly due to the emergence of vaccines, more effective treatments, changes in virus variants and reduced assistance pressure in hospitals. The proportion of patients experiencing ARDS (40%) was similar to other studies [6, 22, 23]. Despite being high, the incidence of ARDS in hospitalized patients is likely underestimated in most studies, as physicians do not always document this diagnosis in the medical records [29]. After ARDS, the most common complications were delirium and renal failure, with one-fifth of the hospitalized older patients developing acute kidney injury. Other cohort studies assessing hospitalized older patients have reported acute kidney injury, which is likely related to tubular injury caused by local and systemic inflammation and immune systems, aggravated by hemodynamic instability [22, 23, 31]. Moreover, underlying chronic kidney disease is frequent among octogenarians, further contributing to an increased risk of developing acute kidney injury during hospitalization.

Older patients are particularly vulnerable to developing severe forms of the disease [31]. The current study further confirms the clinical presentation, severity, and high mortality rate associated with COVID-19 infection in very older patients during the SARS-CoV-2 pandemic. The mortality rate, exceeding 40% in our hospitalized octogenarian group, aligns with the findings reported by two other Spanish cohorts [29, 33]. Notably, mortality was higher in the first wave than in the others, and the mortality rate decreased with the progression of the epidemic waves, likely reflecting the successful use of vaccines (starting massively in March 2021) and more effective treatments as the pandemic advanced. Few studies on the older patients have analyzed clinical presentation, hospital mortality, and their associated factors across different epidemic waves [34, 35]. Only one previous study has specifically focused on octogenarian patients [23].This study, that focused only on the first two epidemic waves and did not analyze risk factors for complications, found a slightly higher overall fatality rate than ours (46.9%), possibly because the study did not include vaccinated patients, which is associated with lower mortality.

Regarding the risk of mortality during hospitalization, this study found that age and elevated levels of CRP and sodium were associated with a higher risk of death. These findings have been consistent with previous studies conducted on older patients [24, 36, 37]. During the hospitalization of COVID-19 patients, both hyponatremia and hypernatremia have been associated with worse prognoses and increased mortality rates [38, 39]. In our study, elevated sodium levels emerged as a risk factor for death and complications, possibly because patients in a more severe condition may experience tachypnea, fever, and dehydration. On the other hand, patients who presented with a cough at the time of hospitalization and those with higher Barthel index scores had a lower risk of death. While no studies specifically analyze cough as a risk factor for mortality in older patients, studies encompassing patients of all ages have yielded contradictory results [40, 41]. Regarding the Barthel index score, other studies have reported similar findings to ours, indicating a higher degree of dependence as a risk factor for mortality [24, 36].

This study was limited by the use of secondary data obtained from medical records, which could introduce information biases related to underreporting certain symptoms or pathologies, in addition to missing information. For the same reason, variables associated with geriatric syndromes (such as malnutrition, falls, or frailty) could not be studied. However, the pre-existing functional status (Barthel index) and delirium were adequately recorded in terms of frequency and quality, allowing their inclusion in our analysis and confirming their significant role in COVID-19 among the older people. Vaccination started late in this study, resulting in a relatively low number of vaccinated patients, limited to the most recent epidemic waves. This is why the effect of vaccines could not be well studied, although it may be related to the improved prognosis observed in the latest epidemic waves, as reflected in our data. Additionally, the heterogeneity in the collection of certain laboratory parameters across different hospitals (with varying units or reference levels) and throughout the different waves posed a challenge for analysis. Therefore, some laboratory parameters that could not be standardized across the databases were excluded from the analysis, precluding their inclusion in the multivariate analysis. It is important to note that our findings exclusively apply to older patients hospitalized with COVID-19 and may not be applicable to older individuals with milder forms of the disease who did not require hospitalization.

Despite these limitations, a substantial number of hospitalized octogenarian patients with COVID-19 were included across different waves of the disease in various hospital centers, yielding robust results. Thus, this study offers valuable insights into the disease among the older people, specifically regarding the characteristics of hospitalized patients, the clinical manifestations of the disease, abnormalities observed in laboratory and radiological assessments, the frequency and types of complications, as well as the in-hospitalmortality rate and prognostic factors, which may help identify older patients who are at risk of developing complications or death during their hospital stay.

## Conclusions

This study describes the clinical characteristics, predictors of complications, and mortality in octogenarian patients hospitalized with COVID-19 across multiple epidemic waves. Our findings provide insight into the disease and its risk factors in older patients, contributing to the development of tailored therapeutic interventions and strategies aimed at reducing mortality and complications from COVID-19 in this patient group.

## Supplementary Information

Below is the link to the electronic supplementary material.

Supplementary file1 (DOCX 56 kb)
