# Correction: Clinical characteristics and predictors of complications and mortality in hospitalized octogenarian patients with COVID-19: an ambispective study

**DOI:** 10.1007/s41999-025-01249-1

**Published:** 2025-06-04

**Authors:** Marta Arroyo-Huidobro, Natàlia Pallarès Fontanet, Cristian Tebé Cordomí, Antonella F. Simonetti, Carlos Pérez-López, Gabriela Abelenda-Alonso, Alexander Rombauts, Isabel Oriol Bermudez, Elisenda Izquierdo, Vicente Díaz-Brito, Gemma Molist, Guadalupe Gómez Melis, Sebastian Videla, Alfons López Soto, Jordi Carratalà, Alejandro Rodriguez-Molinero, On behalf of MetroSud and Divine Study Group, Carlota Gudiol, Judit Aranda-Lobo, Montserrat Sanmartí, Encarna Moreno, Maria C. Alvarez, Ana Faura, Martha González, Paula Cruz, Mireia Colom, Andrea Perez, Laura Serrano, Sebastià Videla, Mireia Besalú, Erik Cobo, Jordi Cortés, Daniel Fernández, Leire Garmendia, Guadalupe Gómez, Pilar Hereu, Klaus Langohr, Núria Pérez-Álvarez, Xavier Piulachs, Natàlia Pallares, Cristian Tebé, Mireia Besalú, Erik Cobo, Jordi Cortés, Daniel Fernández, Klaus Langohr, Núria Pérez-Álvarez, Xavier Piulachs, Guadalupe Gómez

**Affiliations:** 1 Geriatric Unit, Hospital Clinic de Barcelona, C. de Villarroel, 170, 08036 Barcelona, Spain; 2 Biostatistics Support and Research Unit, Germans Trias i Pujol Research Institute and Hospital (IGTP), Badalona, Catalunya Spain; 3 Infectious Diseases Unit, Hospital de la Santa Creu i Sant Pau, Barcelona, Catalunya Spain; 4 Consorci Sanitari Alt Pènedes I Garraf, Area de Recerca, Barcelona, Catalunya Spain; 5 Department of Infectious Diseases, Bellvitge University Hospital, L’Hospitalet de Llobregat, Catalunya Spain; 6 Department of Internal Medicine, Consorci Sanitari Integral, L’Hospitalet de Llobregat, Catalunya Spain; 7 Department of Anaesthesiology, Hospital de Viladecans, Viladecans, Catalunya Spain; 8 Parc Sanitari Sant Joan de Déu, Sant Boi de Llobregat, Spain; 9 Biostatistics Unit of the Bellvitge Biomedical Research Institute (IDIBELL), L’Hospitalet de Llobregat, Catalunya Spain; 10 Department of Statistics and Operations Research, Universitat Politècnica de Catalunya/Barcelonatech, Barcelona, Catalunya Spain; 11 Department of Clinical Pharmacology, Bellvitge University Hospital, L’Hospitalet de Llobregat, Catalunya Spain; 12 Hospital Clinic de Barcelona, Geriatric Unit, Department of Internal Medicine, Institut d’Investigacions Biomèdiques August Pi i Sunyer (IDIBAPS), Barcelona, Catalunya Spain; 13 Consorci Sanitari Alt Penedes-Garraf, Area de Recerca, Barcelona, Catalunya Spain

**Correction: European Geriatric Medicine (2024) 15:1477–1487** 10.1007/s41999-024-01063-1

We have recently noticed a typographical error in the spelling of one of the author’s names.

The correct form of the name is Alejandro Rodriguez-Molinero, with a hyphen between the surnames. In the published version, the name appears incorrectly as Alejandro Rodriguez Molinero (without the hyphen).

Thank you very much for your attention to this matter, and we apologize for any inconvenience caused.

