# Supplementary material for: Clinical characteristics and predictors of complications and mortality in hospitalized octogenarian patients with COVID-19: an ambispective study
Source: Eur Geriatr Med. 2024 Oct 19;15(5):1477–87. doi: 10.1007/s41999-024-01063-1 (PMC11615005; doi:10.1007/s41999-024-01063-1)
Supplement: Supplementary file 1 — Supplementary file1 (DOCX 56 kb) [file 41999_2024_1063_MOESM1_ESM.docx]

# Supplementary Tables

| **Table S1.** Most frequent previous treatments (used by >5% of patients), n (%) | | | | | |
| --- | --- | --- | --- | --- | --- |
|  | **1st wave**  n=730 | **2nd wave**  n=169 | **3rd wave**  n=157 | **5th wave**  n=136 | **Total**  n=1192 |
| **Statins** | 240 (39.5) | 72 (42.9) | 63 (40.1) | 54 (39.7) | 429 (40.2) |
| Atorvastatin | 71 (30.5) | 23 (31.9) | 22 (34.9) | 15 (27.8) | 131 (31.0) |
| Pravastatin | 25 (10.7) | 5 (6.9) | 4 (6.35) | 5 (9.3) | 39 (9.2) |
| Simvastatin | 133 (57.1) | 42 (58.3) | 37 (58.7) | 32 (59.3) | 244 (57.8) |
| **ACEI** | 207 (28.5) | 55 (32.7) | 53 (34.0) | 44 (32.4) | 359 (30.2) |
| Enalapril | 121 (71.6) | 39 (70.9) | 35 (66.0) | 29 (65.9) | 224 (69.8) |
| Lisinopril | 24 (14.2) | 8 (14.5) | 8 (15.1) | 10 (22.7) | 50 (15.6) |
| Other | 22 (13.0) | 7 (12.7) | 9 (17.0) | 5 (11.4) | 43 (13.4) |
| **ARBs** | 159 (21.8) | 39 (23.2) | 29 (18.5) | 39 (28.7) | 266 (22.4) |
| Irbesartan | 5 (3.8) | 3 (7.7) | 1 (3.45) | 0 (0.0) | 9 (3.8) |
| Losartan | 74 (56.5) | 23 (59.0) | 17 (58.6) | 22 (56.4) | 136 (57.1) |
| Olmesartan | 7 (5.34) | 3 (7.7) | 0 (0.0) | 2 (5.1) | 12 (5.0) |
| Telmisartan | 3 (2.3) | 2 (5.1) | 2 (6.9) | 1 (2.56) | 8 (3.4) |
| Valsartan | 34 (26.0) | 6 (15.4) | 5 (17.2) | 10 (25.6) | 55 (23.1) |
| Other | 8 (6.1) | 2 (5.1) | 4 (13.8) | 4 (10.3) | 18 (7.6) |
| **Corticosteroids** | 68 (9.4) | 6 (3.6) | 12 (7.6) | 5 (3.7) | 91 (7.7) |
| Systemic | 47 (7.9) | 18 (10.7) | 14 (8.9) | 11 (8.1) | 90 (8.5) |
| Inhaled | 111 (15.5) | 19 (11.3) | 15 (9.6) | 17 (12.5) | 162 (13.8) |
| **Anticoagulants** | 166 (23.2) | 49 (29.2) | 39 (24.8) | 38 (27.9) | 292 (24.8) |
| DOAC | 68 (57.6) | 28 (57.1) | 23 (59.0) | 26 (68.4) | 145 (59.4) |
| VKA | 48 (40.7) | 20 (40.8) | 10 (25.6) | 12 (31.6) | 90 (36.9) |
| LMWH | 2 (1.7) | 1 (2.04) | 6 (15.4) | 0 (0.00) | 9 (3.7) |
| **Acetylsalicylic acid** | 144 (29.4) | 48 (28.6) | 51 (32.5) | 35 (25.7) | 278 (29.3) |
| **Antidiabetics** | 142 (29.8) | 48 (28.6) | 40 (25.5) | 41 (30.1) | 271 (28.9) |

ACEI, angiotensin-converting-enzyme inhibitors; ARBs, angiotensin receptor blockers; DOAC, direct acting oral anticoagulants; VKA, vitamin K antagonists; LMWH, low molecular weight heparins

| **Table S2.** Laboratory and radiological findings | | | | | | |
| --- | --- | --- | --- | --- | --- | --- |
|  | **N** | **1st wave**  n=730 | **2nd wave**  n=169 | **3rd wave**  n=157 | **5th wave**  n=136 | **Total**  n=1192 |
| **Complete blood count** |  |  |  |  |  |  |
| Hemoglobin g/dL, *mean (SD)* | 1113 | 12.8 (1.98) | 12.5 (2.16) | 12.9 (1.85) | 12.4 (1.97) | 12.7 (1.99) |
| Leukocytes ×10^3^/μL, *mean (SD)* | 1128 | 8.00 (3.92) | 7.84 (3.77) | 6.86 (2.88) | 8.08 (4.73) | 7.84 (3.91) |
| Neutrophils ×10^3^/μL, *mean (SD)* | 1121 | 6.38 (3.53) | 6.29 (4.71) | 5.38 (2.69) | 6.02 (3.60) | 6.20 (3.65) |
| Lymphocytes ×10^3^/μL, *mean (SD)* | 1117 | 1.06 (0.97) | 1.04 (0.79) | 0.92 (0.51) | 1.44 (2.87) | 1.08 (1.30) |
| **Serum biochemistry** |  |  |  |  |  |  |
| Glucose (mg/dL), *mean (SD)* | 965 | 147 (72.9) | 143 (72.3) | 147 (64.9) | 140 (59.3) | 145 (69.9) |
| Creatinine (mg/dL), *mean (SD)* | 1097 | 1.38 (0.93) | 1.40 (0.83) | 1.26 (1.04) | 1.25 (0.75) | 1.35 (0.91) |
| D-dimer (ng/mL), *mean (SD)* | 930 | 2293 (4909) | 1905 (4073) | 1651 (4565) | 1120 (2460) | 1989 (4495) |
| AST U/L, *mean (SD)* | 644 | 40.3 (38.5) | 50.1 (60.7) | 40.3 (32.0) | 32.4 (21.5) | 40.6 (40.0) |
| ALT U/L, *mean (SD)* | 981 | 32.0 (56.1) | 33.4 (40.8) | 28.6 (31.1) | 22.5 (17.9) | 30.6 (47.7) |
| LDH U/L, *mean (SD)* | 778 | 354 (173) | 339 (165) | 314 (121) | 292 (292) | 335 (188) |
| Serum Ferritin (μg/L), *mean (SD)* | 443 | 766 (779) | 885 (697) | 1124 (2945) | 684 (1094) | 842 (1561) |
| CRP (mg/L), *mean (SD)* | 1093 | 115 (90.9) | 104 (82.9) | 102 (83.7) | 95.3 (84.2) | 110 (88.4) |
| Procalcitonin (ng/mL), *mean (SD)* | 246 | 2.55 (15.3) | 0.64 (2.90) | 0.36 (0.81) | 0.64 (2.56) | 0.94 (7.17) |
| Venous lactate (mmol/L), *mean (SD)* | 146 | 2.06 (1.69) | 5.82 (19.1) | 1.52 (0.58) | 1.73 (2.66) | 2.45 (7.43) |
| **Arterial blood gases** |  |  |  |  |  |  |
| Blood gas type, n (%) |  | 376 | 129 | 99 | 108 | 712 |
| Arterial |  | 304 (80.9) | 114 (88.4) | 91 (91.9) | 103 (95.4) | 612 (86.0) |
| Venous |  | 72 (19.1) | 15 (11.6) | 8 (8.08) | 5 (4.63) | 100 (14.0) |
| FiO2 gasometry %, *mean (SD)* | 600 | 26.8 (23.7) | 31.7 (19.3) | 30.4 (16.8) | 27.2 (10.6) | 28.1 (21.0) |
| pH gasometry, *mean (SD)* | 714 | 7.44 (0.06) | 7.44 (0.06) | 7.45 (0.06) | 7.44 (0.06) | 7.44 (0.06) |
| paO2 gasometry (mmHg), Mean (SD) | 746 | 75.5 (37.5) | 79.9 (40.2) | 85.1 (49.3) | 85.2 (35.7) | 78.8 (39.5) |
| pCO2 gasometry (mmHg), *mean (SD)* | 837 | 35.4 (7.62) | 35.9 (7.64) | 35.5 (6.46) | 37.7 (8.35) | 35.8 (7.62) |
| **Chest x-ray** |  |  |  |  |  |  |
| Normal Chest x-ray, n (%) | 1173 | 95 (13.3) | 15 (8.93) | 24 (15.5) | 20 (14.7) | 154 (13.1) |
| Chest x-ray findings, n (%) |  | 686 | 168 | 155 | 136 | 1145 |
| Normal |  | 104 (15.2) | 16 (9.52) | 25 (16.1) | 22 (16.2) | 167 (14.6) |
| Abnormal unilateral |  | 81 (11.8) | 21 (12.5) | 20 (12.9) | 26 (19.1) | 148 (12.9) |
| Abnormal bilateral |  | 501 (73.0) | 131 (78.0) | 110 (71.0) | 88 (64.7) | 830 (72.5) |
| *Chest x-ray pattern type, n (%)* |  |  |  |  |  |  |
| Unilateral interstitial | 818 | 51 (12.0) | 18 (12.3) | 27 (20.6) | 23 (19.8) | 119 (14.5) |
| Bilateral interstitial | 962 | 317 (56.3) | 101 (66.4) | 85 (64.9) | 76 (65.5) | 579 (60.2) |
| Cotton-wool infiltrates | 931 | 105 (19.5) | 11 (7.53) | 5 (3.82) | 5 (4.31) | 126 (13.5) |
| Bilateral interstitial + cotton-wool infiltrates | 936 | 217 (40.0) | 33 (22.4) | 25 (19.1) | 12 (10.3) | 287 (30.7) |
| Pleural effusion | 800 | 30 (7.46) | 6 (3.95) | 5 (3.85) | 2 (1.72) | 43 (5.38) |

SD, standard deviation; PT, prothrombin time; AST, alanine transaminase; ALT, alanine transaminase; LDH, lactate dehydrogenase; CRP, C-reactive protein.

| **Table S3.** Mortality and most frequent complications (presented in >3% of patients), n (%) | | | | | |
| --- | --- | --- | --- | --- | --- |
|  | **1st wave**  n=730 | **2nd wave**  n=169 | **3rd wave**  n=157 | **5th wave**  n=136 | **Total**  n=1192 |
| **Deaths** | 337 (46.2) | 67 (39.6) | 56 (35.7) | 33 (24.3) | 493 (41.4) |
| **Complications** | 353 (48.6) | 88 (52.4) | 72 (45.9) | 56 (41.2) | 569 (47.9) |
| Cardiac complications | 76 (10.5) | 20 (11.9) | 19 (12.1) | 11 (8.09) | 126 (10.6) |
| Heart failure | 42 (5.8) | 14 (8.3) | 10 (6.37) | 7 (5.2) | 73 (6.1) |
| Atrial fibrillation | 32 (94.1) | 9 (90.0) | 10 (100) | 6 (100) | 57 (95.0) |
| Pulmonary complications | 33 (4.5) | 14 (8.3) | 4 (2.6) | 9 (6.6) | 60 (5.1) |
| ARDS | 291 (40.0) | 85 (50.9) | 81 (51.6) | 62 (45.6) | 519 (43.7) |
| Renal failure | 157 (21.6) | 31 (18.5) | 25 (15.9) | 15 (11.0) | 228 (19.2) |
| Delirium* | 142 (19.5) | 28 (16.7) | 22 (14.0) | 16 (11.8) | 208 (17.5) |
| Nosocomial infection | 22 (3.03) | 10 (5.9) | 10 (6.4) | 6 (4.4) | 48 (4.0) |
| Catheter bacteremia | 3 (15.8) | 3 (30.0) | 0 (0.0) | 2 (33.3) | 8 (17.8) |
| Urinary tract infection | 17 (81.0) | 6 (60.0) | 6 (60.0) | 4 (66.7) | 33 (70.2) |

ARDS, acute respiratory distress syndrome

*Based on the diagnosis recorded in the discharge report at the end of hospitalization (*delirium* or *acute confusional syndrome*)

**Table S4.** Stratified analysis of mortality and any complication: Comparison between the first wave and subsequent waves

| **Mortality** | N | n | % | IC 95% |
| --- | --- | --- | --- | --- |
| Wave 1 | 425 | 186 | 43.76 | (38.99, 48.63) |
| Wave 2-5 | 391 | 131 | 33.5 | (28.84, 38.42) |
|  |  |  |  |  |
| **Any complication** |  |  |  |  |
| Wave 1 | 423 | 200 | 47.28 | (42.44, 52.16) |
| Wave 2-5 | 390 | 155 | 39.74 | (34.85, 44.79) |
|  |  |  |  |  |
| **Delirium*** |  |  |  |  |
| Wave 1 | 423 | 89 | 21.04 | (17.25, 25.24) |
| Wave 2-5 | 390 | 54 | 13.85 | (10.58, 17.68) |
|  |  |  |  |  |
| **Renal complications**^§^ |  |  |  |  |
| Wave 1 | 423 | 93 | 21.99 | (18.13, 26.24) |
| Wave 2-5 | 390 | 64 | 16.41 | (12.87, 20.47) |
|  |  |  |  |  |
| **Cardiac complications**^†^ |  |  |  |  |
| Wave 1 | 423 | 53 | 12.53 | (9.53, 16.07) |
| Wave 2-5 | 390 | 46 | 11.79 | (8.77, 15.42) |

* Based on the diagnosis recorded in the discharge report at the end of hospitalization (*delirium* or *acute confusional syndrome*)

^§^ Renal failure

^†^ Heart failure, atrial fibrillation

| **Table S5.** Multivariate analysis assessing the risk of delirium | | | | |
| --- | --- | --- | --- | --- |
|  | **Odds Ratio** | **Std. Error** | **95% CI** | ***P*-value** |
| ***Sociodemographic and comorbidity predictors*** | | | | |
| Age | 1.23 | 0.15 | 0.97 – 1.57 | 0.087 |
| Sex (Female) | 0.61 | 0.13 | 0.40 – 0.93 | **0.023** |
| Barthel index | 0.94 | 0.04 | 0.86 – 1.02 | 0.110 |
| High Blood Pressure | 0.73 | 0.19 | 0.44 – 1.23 | 0.230 |
| Dyslipidemia | 1.04 | 0.22 | 0.69 – 1.57 | 0.856 |
| Chronic Obstructive Pulmonary Disease | 1.04 | 0.26 | 0.64 – 1.68 | 0.862 |
| Dementia | 1.86 | 0.49 | 1.10 – 3.10 | **0.019** |
| Corticosteroids | 1.39 | 0.36 | 0.82 – 2.30 | 0.211 |
| Hearth Failure | 1.00 | 0.25 | 0.60 – 1.60 | 0.985 |
| ***Clinical predictors*** | | | | |
| Cough | 0.73 | 0.14 | 0.50 – 1.05 | 0.068 |
| Dyspnea | 1.13 | 0.23 | 0.78 – 1.65 | 0.516 |
| Abnormal unilateral chest x-ray | 0.53 | 0.20 | 0.25 – 1.10 | 0.092 |
| Abnormal bilateral chest x-ray | 0.61 | 0.16 | 0.36 – 1.04 | 0.061 |
| ***Radiological and laboratory predictors*** | | | | |
| Total leukocytes | 1.05 | 0.28 | 0.61 – 1.75 | 0.846 |
| D- Dimer | 1.00 | 0.00 | 1.00 – 1.00 | 0.154 |
| CRP | 1.02 | 0.01 | 1.00 – 1.04 | **0.027** |
| Sodium | 1.40 | 0.21 | 1.04 – 1.89 | **0.026** |
| Basal creatinine | 1.13 | 0.21 | 0.79 – 1.64 | 0.510 |

Std, standard; 95% CI, 95% confidence interval; CRP, C-reactive protein

Age was evaluated as 5 years increments. Total leukocytes, CRP, sodium, creatinine were evaluated as 10 units increments. D- dimer was evaluated as 50 units increments.

| **Table S6.** Multivariate analysis assessing the risk of cardiac complications | | | | |
| --- | --- | --- | --- | --- |
|  | **Odds Ratio** | **Std. Error** | **95% CI** | ***P*-value** |
| ***Sociodemographic and comorbidity predictors*** | | | | |
| Age | 1.19 | 0.17 | 0.90 – 1.56 | 0.222 |
| Sex (Female) | 1.27 | 0.30 | 0.79 – 2.03 | 0.326 |
| Barthel index | 0.96 | 0.05 | 0.87 – 1.06 | 0.411 |
| High Blood Pressure | 0.75 | 0.23 | 0.42 – 1.39 | 0.340 |
| Dyslipidemia | 1.14 | 0.27 | 0.71 – 1.83 | 0.592 |
| Chronic Obstructive Pulmonary Disease | 0.72 | 0.21 | 0.40 – 1.25 | 0.252 |
| Dementia | 0.70 | 0.23 | 0.36 – 1.32 | 0.281 |
| Corticosteroids | 1.59 | 0.46 | 0.89 – 2.77 | 0.109 |
| Heart Failure | 2.70 | 0.66 | 1.67 – 4.35 | **<0.001** |
| ***Clinical predictors*** | | | | |
| Cough | 1.33 | 0.30 | 0.85 – 2.10 | 0.217 |
| Dyspnea | 2.56 | 0.61 | 1.62 – 4.14 | **<0.001** |
| Abnormal unilateral chest x-ray | 1.53 | 0.73 | 0.61 – 4.05 | 0.373 |
| Abnormal bilateral chest x-ray | 1.31 | 0.52 | 0.64 – 3.06 | 0.494 |
| ***Radiological and laboratory predictors*** | | | | |
| Total leukocytes | 1.08 | 0.34 | 0.57 – 1.94 | 0.814 |
| D- Dimer | 1.00 | 0.00 | 1.00 – 1.00 | 0.606 |
| CRP | 1.00 | 0.01 | 0.98 – 1.03 | 0.725 |
| Sodium | 0.88 | 0.18 | 0.59 – 1.29 | 0.533 |
| Basal creatinine | 0.81 | 0.18 | 0.53 – 1.25 | 0.333 |

Std, standard; 95% CI, 95% confidence interval; CRP, C-reactive protein

Age was evaluated as 5 years increments. Total leukocytes, CRP, sodium, creatinine were evaluated as 10 units increments. D- dimer was evaluated as 50 units increments.

| **Table S7.** Multivariate analysis for risk of renal complications | | | | |
| --- | --- | --- | --- | --- |
|  | **Odds Ratio** | **Std. Error** | **95% CI** | ***P*-value** |
| ***Sociodemographic and comorbidity predictors*** | | | | |
| Age | 1.08 | 0.14 | 0.83 – 1.39 | 0.574 |
| Sex (Female) | 0.63 | 0.14 | 0.40 – 0.98 | **0.041** |
| Barthel index | 0.93 | 0.04 | 0.85 – 1.01 | 0.091 |
| Diabetes Mellitus | 1.86 | 0.40 | 1.22 – 2.82 | **0.004** |
| High Blood Pressure | 1.46 | 0.45 | 0.82 – 2.77 | 0.222 |
| Dyslipidemia | 1.12 | 0.24 | 0.73 – 1.71 | 0.614 |
| Obesity | 1.00 | 0.23 | 0.64 – 1.56 | 0.990 |
| Chronic Obstructive Pulmonary Disease | 1.00 | 0.25 | 0.61 – 1.61 | 0.986 |
| Dementia | 0.77 | 0.23 | 0.43 – 1.36 | 0.381 |
| Corticosteroids | 0.98 | 0.26 | 0.57 – 1.63 | **0.930** |
| Heart Failure | 1.67 | 0.39 | 1.05 – 2.63 | **0.029** |
| ***Clinical predictors*** | | | | |
| Cough | 0.77 | 0.14 | 0.54 – 1.10 | 0.148 |
| Dyspnea | 1.16 | 0.21 | 0.81 – 1.67 | 0.422 |
| Abnormal unilateral chest x-ray | 0.96 | 0.33 | 0.48 – 1.90 | 0.902 |
| Abnormal bilateral chest x-ray | 0.74 | 0.20 | 0.44 – 1.29 | 0.277 |
| ***Radiological and laboratory predictors*** | | | | |
| Total leukocytes | 0.97 | 0.27 | 0.56 – 1.65 | 0.921 |
| D- Dimer | 1.00 | 0.00 | 1.00 – 1.00 | 0.351 |
| CRP | 1.02 | 0.01 | 1.00 – 1.05 | **0.091** |
| Sodium | 1.15 | 0.18 | 0.84 – 1.58 | **0.392** |
| Basal creatinine | 8.26 | 1.92 | 5.32 – 13.22 | **<0.001** |

Std, standard; 95% CI, 95% confidence interval; CRP, C-reactive protein

Age was evaluated as 5 years increments. Total leukocytes, CRP, sodium, and creatinine were evaluated as 10 units increments. D- dimer was evaluated as 50 units increments.

| **Table S8. S**ummary of predictors for death and complications | | | | | |
| --- | --- | --- | --- | --- | --- |
|  | **Death** | **Any complication** | **Delirium*** | **Cardiac complications**^†^ | **Renal Complications**^§^ |
| **Risk factor** | Age, dyspnea, abnormal bilateral chest x-ray, elevated levels of CRP, and sodium | Age, history of DM, heart failure, dyspnea, and elevated creatinine levels | Dementia and elevated sodium levels | Heart failure and dyspnea | DM, heart failure, and elevated creatinine levels |
| **Protective Factor** | Higher barthel index, cough | Higher barthel index, cough | Female sex |  |  |

CRP, C-reactive protein; DM, Diabetes Mellitus

* Based on the diagnosis recorded in the discharge report at the end of hospitalization (*delirium* or *acute confusional syndrome*)

^†^ Heart failure, atrial fibrillation

^§^ Renal failure
